# Dual engineered bacteria improve inflammatory bowel disease in mice

**DOI:** 10.1007/s00253-024-13163-w

**Published:** 2024-05-13

**Authors:** Yong-Qi Wu, Zhen-Ping Zou, Ying Zhou, Bang-Ce Ye

**Affiliations:** 1 Laboratory of Biosystems and Microanalysis, State Key Laboratory of Bioreactor Engineering, East China University of Science and Technology, Shanghai, 200237 China; 2 Institute of Engineering Biology and Health, Collaborative Innovation Center of Yangtze River Delta Region Green Pharmaceuticals, College of Pharmaceutical Sciences, Zhejiang University of Technology, Hangzhou, Zhejiang 310014 China

**Keywords:** Engineered bacteria, Inflammatory bowel disease, *E.coli* Nissle 1917, Anti-TNF-α nanobody, IL-10

## Abstract

**Abstract:**

Currently, there are many different therapies available for inflammatory bowel disease (IBD), including engineered live bacterial therapeutics. However, most of these studies focus on producing a single therapeutic drug using individual bacteria, which may cause inefficacy. The use of dual drugs can enhance therapeutic effects. However, expressing multiple therapeutic drugs in one bacterial chassis increases the burden on the bacterium and hinders good secretion and expression. Therefore, a dual-bacterial, dual-drug expression system allows for the introduction of two probiotic chassis and enhances both therapeutic and probiotic effects. In this study, we constructed a dual bacterial system to simultaneously neutralize pro-inflammatory factors and enhance the anti-inflammatory pathway. These bacteria for therapy consist of *Escherichia coli* Nissle 1917 that expressed and secreted anti-TNF-α nanobody and IL-10, respectively. The oral administration of genetically engineered bacteria led to a decrease in inflammatory cell infiltration in colon and a reduction in the levels of pro-inflammatory cytokines. Additionally, the administration of engineered bacteria did not markedly aggravate gut fibrosis and had a moderating effect on intestinal microbes. This system proposes a dual-engineered bacterial drug combination treatment therapy for inflammatory bowel disease, which provides a new approach to intervene and treat IBD.

**Key points:**

• *The paper discusses the effects of using dual engineered bacteria on IBD*

• *Prospects of engineered bacteria in the clinical treatment of IBD*

**Graphical Abstract:**

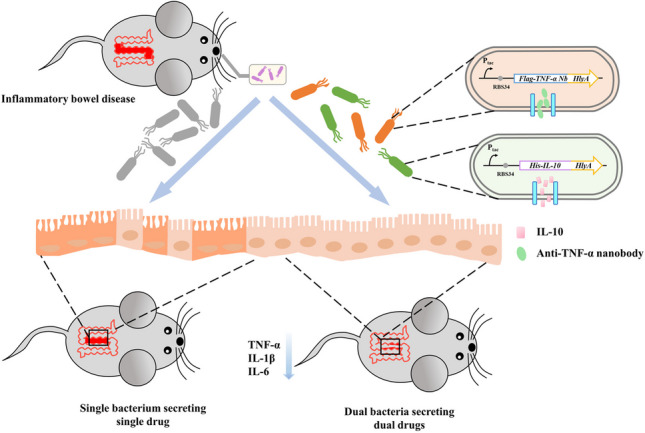

**Supplementary information:**

The online version contains supplementary material available at 10.1007/s00253-024-13163-w.

## Introduction

Inflammatory bowel disease (IBD) is a serious gastrointestinal disorder that typically presents with symptoms, such as diarrhea, abdominal discomfort, bloody stools and weight loss. Persistent inflammation may elevate the likelihood of developing severe conditions, including colorectal cancer (Chen et al. 2023). The main therapeutic agents for IBD currently include chemotherapeutic agents (aminosalicylates) and protein drugs (infliximab and adalimumab). These drugs are typically administered systemically via intravenous injection to treat IBD (Chen et al. 2023). It is worth noting that intravenous administration of protein drugs is prone to adverse reactions, with a higher incidence of allergic reactions than that oral administration (Hanauer et al. 2002; Lichtenstein et al. 2006). Oral administration of engineered live bacterial therapeutics can partially overcome these disadvantages (Harimoto et al. 2022; Li et al. 2021). Praveschotinunt et al. (2019) orally administered engineered *Escherichia coli* Nissle 1917 (EcN) that secreted fibrous matrices to mice with dextran sodium sulfate (DSS)-induced colitis, which effectively promoted gut epithelial integrity in situ. The daily oral administration of *Lactobacillus* secreting anti-mTNF nanobodies achieved targeted delivery to the colon, resulting in a significant reduction in inflammation in mice with DSS-induced chronic colitis (Vandenbroucke et al. 2010). A distinct class of immunoglobulins lacking light chains, referred to as nanobody (Nb) or VHHs (variable heavy chain), have been identified in shark and camel species (Verhaar et al. 2021). Their compact size (~ 15 kDa) confers enhanced tissue penetration capabilities. The intragastric administration of *Lactobacillus lactis* secreting interleukin-10 (IL-10) led to a substantial 50% reduction in colitis among DSS-treated mice and effectively prevented the onset of colitis in IL-10^−/−^ mice (Steidler et al. 2000). Interleukin-10 (IL-10), a multifaceted cytokine, exerts diverse effects on cellular growth, differentiation, inflammation, and immune responses. It plays a crucial role in inflammation and immunosuppression and is recognized as an important factor in tumor development, infection control, organ transplantation, hematopoietic system regulation, and cardiovascular function (Moore et al. 2001). However, the majority of such studies focus on generating a single therapeutic protein using bacterial strains (Steidler et al. 2000; Vandenbroucke et al. 2010). This approach may lead to drug resistance and result in no significant therapeutic effect after long-term use of the drug (Roda et al. 2016). When compared with monotherapy, the combined administration of guselkumab and golimumab, targeting multiple biological pathways, demonstrated superior efficacy in the treatment of active ulcerative colitis (Feagan et al. 2023). Consequently, we chose anti-TNF-α nanobody and interleukin-10 (IL-10) as therapeutic proteins for the management of IBD.

*E. coli* Nissle 1917 (EcN) exhibits promising attributes, including facile genetic manipulation and probiotic characteristics, making it an ideal candidate for biotherapeutic applications. This non-pathogenic strain, serotype O6:K5:H1, exhibits antagonistic activities against various gut pathogens. Moreover, EcN engineering benefits from the availability of comprehensive molecular biology toolboxes (Kruis et al. 2004; Lynch et al. 2022; Sonnenborn 2016; Tan et al. 2020). Notably, EcN remains genetically stable and resistant to transformation by tox-phages found in enterohemorrhagic *E. coli* strains (Sonnenborn et al. 2009). Previous studies have demonstrated its production of defensins, cathelicidin, and calprotectin along with anti-inflammatory effects like inhibition of IL-6 and TNF-α (Choudhary et al. 2020; Grabig et al. 2006; Kai-Larsen et al. 2010; Wehkamp et al. 2004).

Here, we constructed two genetically engineered *E. coli* Nissle 1917 (EcN) strains capable of expressing and secreting anti-TNF-α nanobody and IL-10, respectively. The genetically modified bacteria effectively suppressed colonic inflammation induced by dextran sulfate sodium (DSS), as demonstrated by reduced colon shortening, lower disease activity index, and modulation of pro-inflammatory cytokines expression in colon tissues. More interestingly, architectural changes, inflammatory cell infiltrations, and epithelial injuries were reduced when treated with EcN-TNFaNb or/and EcN-IL10. Additionally, the administration of engineered bacteria did not induce gut fibrosis. Furthermore, the 16 S rRNA results verified that engineered bacteria could modulate the gut microbiome in DSS-induced IBD mice. Considering that the certain therapeutic efficacy achieved through the combination of two engineered bacteria, we foresee a promising future for engineered live bacterial therapeutics in treating IBD. This will promote the research and clinical application of novel drugs in this field.

## Materials and methods

### Bacterial strains and culture conditions

For plasmid construction and bacterial activation, the engineered bacteria (Supplemental Table S1) were cultured in lysogeny broth (LB) media with the respective antibiotics (50 µg/mL streptomycin or 50 µg/mL kanamycin) for 12 h at 37 °C.

### Gene cloning and soluble expression of anti-TNF-α nanobody and IL-10 protein

We obtained the amino acid sequences of mouse anti-TNF-α nanobody and mouse IL-10 from literature reports (Vandenbroucke et al. 2010) and the NCBI website (www.ncbi.nlm.nih.gov), respectively (Supplemental Table S2). The respective DNA sequences were synthesized by Shanghai Ruimian Biotechnology Co., Ltd. (China) using PCR technology. Subsequently, the gene sequences for anti-TNF-α nanobody and IL-10, along with a sequence for a 6xHis tag, were inserted into the protein expression backbone vector pET-28a (www.snapgene.com/plasmids/pet_and_duet_vectors_(novagen)/pET-28a(%2B)). Next, the engineered plasmids were introduced into *E. coli* BL21 individually to create IPTG (isopropyl β-D-thiogalactoside)-inducible expression strains. The recombinant proteins were obtained following the previously described protocols (Zou et al. 2023). In short, after IPTG induction, the BL21 cells were collected from the cultures via centrifugation and then resuspended in PBS buffer, followed by ultrasonic cell disruption to obtain the product described as “whole protein.” Subsequently, the supernatant of the samples were collected through centrifugation and the purified products were obtained by purifying the supernatant using nickel columns (Smart Lifescience, Changzhou, China). Whole protein, supernatant, and purified products were analyzed by sodium dodecyl sulfate polyacrylamide gel electrophoresis (SDS-PAGE).

### Cell experiments and cytokine detection

RAW264.7 macrophage cells (Oh et al. 2006) were cultured at 37℃ in a 5% CO_2_ environment. The cells were maintained in Dulbecco’s modified Eagle medium (DMEM) with high glucose and 10% fetal bovine serum (FBS). RAW264.7 cells were utilized to assess the effects of anti-TNF-α nanobody and IL-10. Cells were harvested when they reached approximately 70–80% confluence in a 25 cm^2^ culture flask containing 5 mL of medium.

The suspension was then collected by centrifugation at 800 rcf (relative centrifugal force) for 5 min. Following centrifugation, the cells were resuspended in DMEM medium and adjusted to a concentration of 5 × 10^4^ cells/mL. An 800 µL of suspension was added to each well. To the wells of the culture plate, either an anti-TNF-α nanobody or IL-10 was added at a final concentration of 20 µg/mL. The cells were pre-incubated for 2 h before being stimulated with 100 µg/mL lipopolysaccharide (LPS) for 14 h. To determine the levels of cytokines, cytokine detection kits from NEOBIOSCIENCE (Shenzhen, China) were used, following the instructions.

### Construction of constitutive and inducible engineered bacteria

The plasmids were constructed in *E. coli* strain DH5α following standard procedures. The high-strength constitutive promoter P_*tac*_ was used to control the expression of therapeutic proteins in the development of constitutive plasmids containing the pSC101 replication origin (Zou et al. 2023). The sequences of 3´end of the anti-TNF-α nanobody gene and the IL-10 gene were fused with the DNA sequence for the secretory peptide encoded by gene *HlyA*, and the anti-TNF-α nanobody gene and the IL-10 gene were fused with the sequences for the Flag-tag and the His-tag, respectively.

To construct inducible plasmids, we integrated *thsS/R* from *E. coli* Nissle 1917, whose products can sense and respond to thiosulfate (Daeffler et al. 2017), under the control of promoters P_*j123104*_ and P_*j123100*_ into a low-copy plasmid with pSC101 as its replication origin. In addition, we utilized promoter P_*phsA*_, which responds to thiosulfate via ThsR, to regulate the expression of drug proteins. Subsequently, we transformed these plasmids into the engineered *E. coli* strain EcN where α-hemolysin secretion system genes (*hlyB* and *hlyD*) were integrated into the chromosome under the control of a constitutive promoter P_*j23104*_.

### Analysis of expression and secretion of anti-TNF-α nanobody and IL-10 in engineered bacteria by western blotting and enzyme linked immune sorbent assay (ELISA)

To assess the expression and secretion of anti-TNF-α nanobody and IL-10 in EcN-TNFαNb and EcN-IL10 strains, the strains were cultured in LB medium with respective antibiotics until reaching OD_600_ ≈ 0.6 and then incubated with shaking for durations of 6, 8, 10, and 12 h. Then, the supernatant of culture (LB medium supernatant) was collected by centrifugation. The engineering bacterial cells in the precipitate were resuspended with PBS and subjected to ultrasonic cell fragmentation to obtain “whole protein.” Finally, the “whole protein supernatant” was collected by centrifugation of the whole protein.

To assess the expression and secretion of anti-TNF-α nanobody and IL-10 in thio-EcN-TNFαNb and thio-EcN-IL10 induced by varying concentrations of thiosulfate, the strains were cultured in LB medium with respective antibiotics until reaching the logarithmic growth phase (OD_600_ ≈ 0.6), followed by a 10-h incubation with different concentrations of thiosulfate (0, 125, 250, and 500 µM) under shaking conditions. Then, the supernatant of culture (LB medium supernatant) was collected by centrifugation. The engineering bacterial cells in the precipitate were resuspended with PBS and subjected to ultrasonic cell fragmentation to obtain “whole protein.” Finally, the “whole protein supernatant” was collected by centrifugation of the whole protein.

Culture supernatant, “whole protein” and “supernatant” were analyzed by gel electrophoresis and subsequently transferred onto a polyvinylidene fluoride (PVDF) membrane for western blotting experiments, following the established protocol (Zou et al. 2023).

A standard curve was generated using purified anti-TNF-α nanobody and IL-10 protein. The anti-TNF-α nanobody protein was diluted to 25, 12.5, 6.25, 3.125, 1.5625, and 0 ng/mL, while the IL-10 protein was diluted to 50, 25, 12.5, 6.25, 3.125, 1.5625, and 0 ng/mL respectively. ELISA experiments were performed according to the previously described protocol (Zou et al. 2023).

### Animal experiments

Male C57BL/6 mice aged 6–8 weeks were procured from Shanghai Model Organisms Center, Inc. (China). They were adapted for one week at 20–22℃ with a 12-h light/12-h dark cycle. All animal experiments strictly followed the Guidelines for the Care and Use of Laboratory Animals established by East China University of Science and Technology. Approval was obtained from the Animal Ethics Committee of the university prior to the commencement of any experiments.

The strains were cultured at 37℃ for 12 h until they reached an OD_600_ of approximately 1.3. Then, the bacteria were collected and resuspended in PBS. The EcN-TNFαNb + EcN-IL10 (PBS, DSS+) group involved culturing the two strains individually and subsequently combining them in a 1:1 ratio. Subsequently, the mice were randomly allocated into six distinct groups, with four mice per group: non-colitis (PBS, DSS^−^), colitis (PBS, DSS^+^), EcN (consisting of the a-hemolysin secretion system) (PBS, DSS^+^), EcN-TNFαNb (PBS, DSS^+^), EcN-IL10 (PBS, DSS^+^), and EcN-TNFαNb + EcN-IL10 (PBS, DSS^+^). On days one and two of the experimental period, each mouse received either PBS or gavage with 5 × 10^9^ colony-forming units (CFUs) of either EcN alone or combined with EcN-TNFαNb, EcN-IL10, or both via oral administration. Starting from day zero to day four consecutively thereafter (day 0–day 4), all mice except those in non-colitis group drank water containing 4% dextran sulfate sodium (DSS) (MW = 36,000–50,000, MP Biomedicals, Irvine, CA, USA). The weights of mice were recorded every day. Seven days after administration, the mice were euthanized, and the lengths of their large intestines were recorded. The section nearest the rectum was fixed in paraformaldehyde for 24 h at room temperature, followed by hematoxylin and eosin staining (HE staining). The middle section was used for cytokines detection. The section closer to the cecum was also stored at -80℃ for future use. Cytokine concentrations were determined using ELISA kits from Sangon Biotech (Shanghai, China).

### Tissue sections

The paraffin-embedded samples were stained with both hematoxylin and eosin (HE staining), as well as Masson trichrome staining solution (Zou et al. 2023). The stained samples were then captured by an inverted microscope.

### Bacterial 16 S rRNA gene sequencing

On the 5th day following administration, fecal samples were collected, promptly frozen at -80℃, and then shipped to Sangon Biotech (Shanghai, China) Co., Ltd., for further analysis, as detailed in the project identification number PRJNA1012370.

### Statistical analysis

Statistical analysis and graphical representation of the data were performed using GraphPad Prism 8 software (GraphPad Inc., Boston, MA, USA). The results were expressed as the mean ± SEM. Statistical significance between groups was determined using a one-way ANOVA test (non-significant = n.s.>0.05, *P ≤ 0.05, **P ≤ 0.01, ***P ≤ 0.001, ****P ≤ 0.0001).

### Data availability

All data presented in this paper will be made available by the corresponding author upon request.

## Results

### Characterization of anti-TNF-α nanobody and IL-10 in vitro

**Fig. 1:**
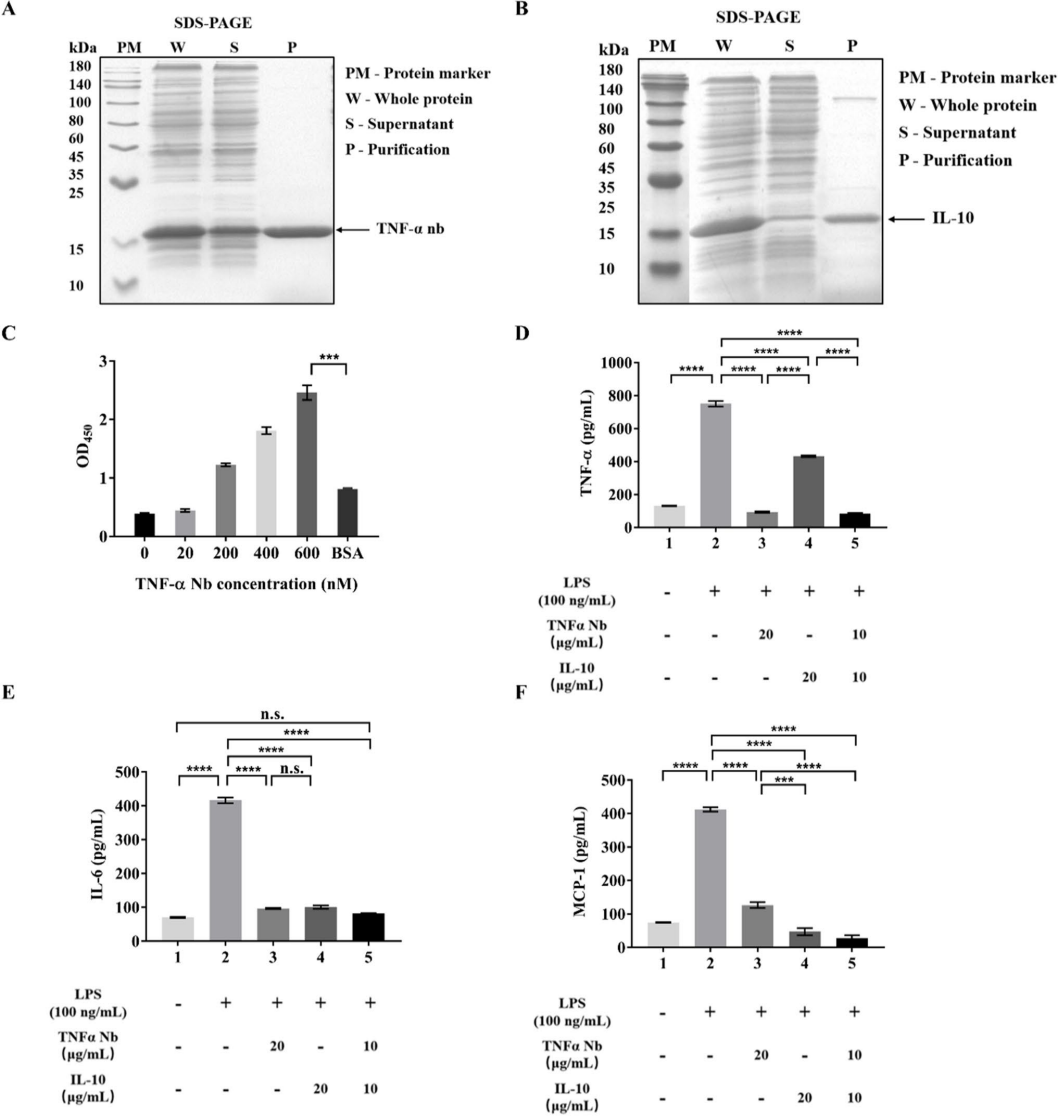
Characterization of anti-TNF-α nanobody and IL-10 in vitro. **A-B** Anti-TNF-α nanobody and IL-10 expression levels in “W,” “S,” and “P” were detected by subjecting *E. coli* BL21 (DE3) bearing anti-TNF-α nanobody and IL-10-gene expressing plasmids to SDS-PAGE after IPTG induction and ultrasonication. The BL21 cells induced by IPTG and collected from the cultures via centrifugation were resuspended in PBS buffer, followed by ultrasonic cell disruption to obtain the product described as “whole protein” (W). Subsequently, the supernatant (S) of the samples were collected through centrifugation, and the purified products (P) were obtained by purifying the supernatant using nickel columns. **C** The specificity and affinity of the anti-TNF-α nanobody to mouse TNF-α were detected by ELISA. **D-F** The effects of drug proteins on the release of cytokines in the LPS-induced RAW264.7. Statistical significance was determined by one-way ANOVA test; *P ≤ 0.05, **P ≤ 0.01, ***P ≤ 0.001, ****P ≤ 0.0001

The anti-TNF-α nanobody and IL-10 genes were obtained from a commercial source and then cloned into a pET28-a plasmid. Successful soluble expression of both the anti-TNF-α nanobody and IL-10 proteins were achieved in the *E. coli* BL21 strain (Fig. 1A–B). SDS-PAGE was used to confirm proper expression of anti-TNF-α nanobody and IL-10. The production levels of the nanobodies and IL-10 were measured at 228.6 µg/mL and 171.7 µg/mL by the BCA kit (Beyotime, Shanghai, China).

We subsequently characterized the specificity and affinity of the antibody to TNF-α. The ELISA results demonstrated the specific binding of the anti-TNF-α nanobody to TNF-α. (Fig. 1C). In the affinity assay, we observed a dose-dependent interaction between the antigen (mouse TNF-α) and the anti-TNF-α nanobody (Fig. 1C). These findings indicate that the anti-TNF-α nanobody exhibits high specificity and affinity.

We assessed the efficacy of the anti-TNF-α nanobody and IL-10 in mitigating inflammation through cellular experiments. RAW264.7 macrophages were stimulated with LPS to establish an inflammation model. ELISA was then employed to measure the secretion of cytokines (Supplemental Fig. S1). In comparison to the untreated control group, the RAW264.7 inflammation model showed a significant increase in secretion levels of TNF-α, IL-6, and MCP-1 (*P* ≤ 0.0001). The purified anti-TNF-α nanobody and IL-10 effectively reduced the cytokine levels, demonstrating significant differences compared with the model group (*P* ≤ 0.0001) (Fig. 1D–F). Our findings suggest that the two drug proteins had a certain alleviating effect on LPS-induced inflammation within RAW264.7 cells; furthermore, their combined effect was superior to either protein alone.

### Construction of the engineered EcN for secreting anti-TNF-α nanobody and IL-10 proteins

**Fig. 2:**
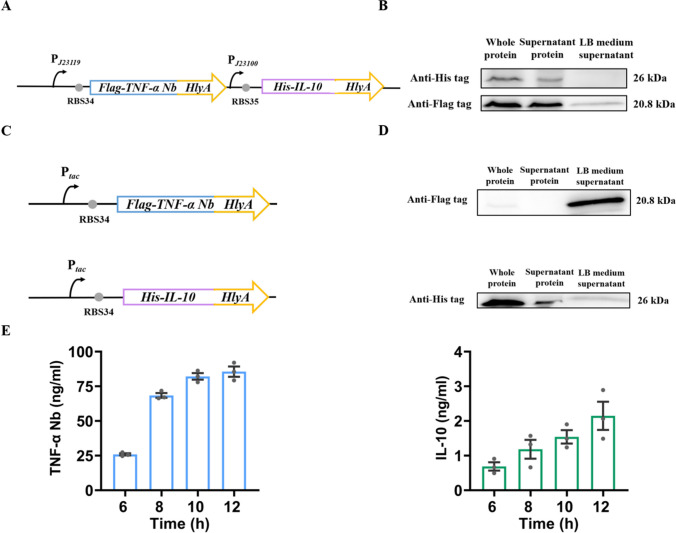
Characterization of constitutive engineered bacteria. **A** Gene route design of constitutive engineered bacteria EcN-TNF-αNb-IL10. **B** Western blot detection results of TNF-α Nb and IL-10 secretion by EcN-TNFaNb-IL10 strain. **C** Gene route design of constitutive engineered bacteria EcN-TNF-α Nb (top) and EcN-IL10 (bottom). **D** Western blot detection results of TNF-α Nb (top) and IL-10 (bottom) secretion. **E** Detection of TNF-α Nb (left) and IL-10 (right) secretion in culture medium by ELISA. EcN-TNFαNb and EcN-IL10 culture supernatant were collected by centrifugation for ELISA analysis at 6, 8, 10, and 12 h. For (**B**) and (**D**), after 12 h of culture, the supernatant (“LB medium supernatant”) was collected from the culture by centrifugation. Separated cell pellets were sonicated for release of whole protein (“whole protein”). Finally, sample supernatants from the samples (“Supernatant protein”) were obtained by separating cell debris through centrifugation

To develop engineered bacteria capable of simultaneous secretion of anti-TNF-α nanobodies and IL-10, we integrated the genes encoding an anti-TNF-α nanobody and IL-10 into a low copy plasmid with the pSC101 origin of replication. The expression of anti-TNF-α nanobody and IL-10 was controlled by constitutive promoters P_*J23119*_ and P_*J23100*_, respectively. It is worth noting that low copy number plasmids usually lead to lower metabolic burden in bacteria and demonstrate greater genetic stability and strong promoters can provide higher protein levels (Barger et al. 2021). To enhance secretion efficiency, we fused the drug protein gene with the DNA sequence of the C-terminus secretory peptide from the *HlyA* gene. In addition, we attached the sequence for a Flag-tag and a His-tag to the 5´ end of the anti-TNF-α nanobody gene and the IL-10 gene, respectively. This resulted in the generation of the pWT-TNFaNb-IL10 plasmid which was subsequently transformed into EcN-hlyBD (containing the α-hemolysin secretion system). The resulting bacteria were named EcN-TNFaNb-IL10 (Fig. 2A). However, Western blotting analysis revealed that IL-10 could not be detected in the culture medium of the EcN-TNFaNb-IL10 strain (Fig. 2B), possibly due to competition within the secretion system leading to significantly reduced secretion efficiency for one of these proteins.

Therefore, in the subsequent experiment, we generated two bacterial strains capable of expressing and secreting anti-TNF-α Nb and IL-10, respectively. The anti-TNF-α nanobody gene was integrated into a low copy plasmid with the pSC101 origin of replication, and its expression was conveyed by a constitutive promoter P_*tac*_ promoter. Subsequently, we fused the anti-TNF-α Nb protein gene with the sequence of *HlyA* for its encoded secretory peptide at its 3´end to create pWT-TNFaNb. This plasmid was then transformed into EcN-hlyBD (containing the α-hemolysin secretion system), resulting in the generation of EcN-TNFaNb bacteria. Similarly, we obtained the EcN-IL10 strain (Fig. 2C). Importantly, integration of these drug protein genes did not induce significant toxicity or inhibit growth of the bacterial EcN strains (Supplemental Fig. S2). ELISA and Western blot analysis confirmed that both anti-TNF-α nanobody and IL-10 were detectable in the culture medium of EcN-TNFαNb and EcN-IL10 strains over a time-dependent period from 6 to 12 h (Fig. 2D–E). Standard curves were presented in the Supplemental Fig. S3. In summary, our study successfully establishes a robust platform for sustained expression of therapeutic drugs in EcN.

### Engineered EcN ameliorates disease activity in mice with DSS-induced colitis

**Fig. 3:**
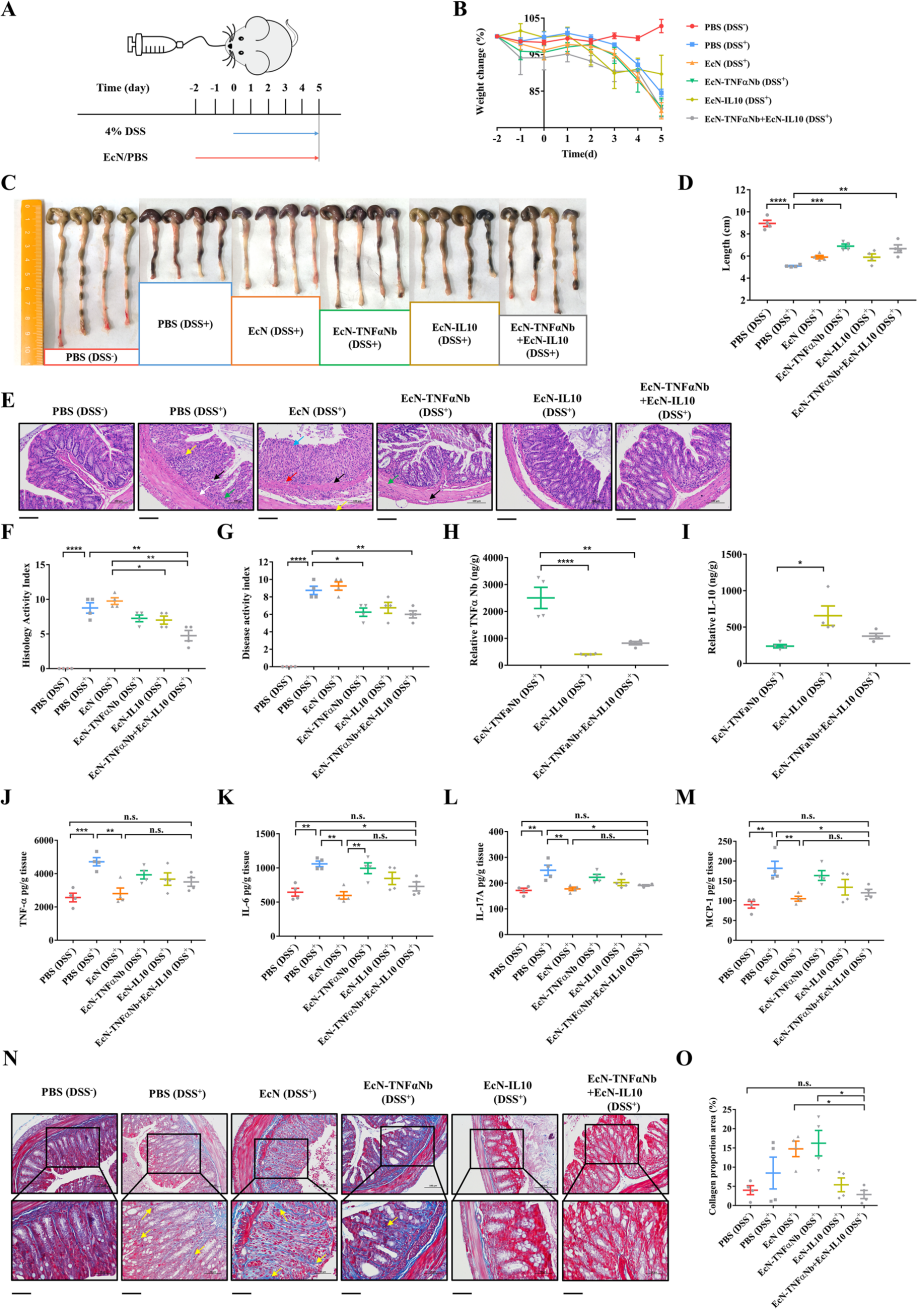
Effects of engineered bacteria on ameliorating mice. **A** Schematic of the administration schedule. PBS or the engineered bacteria (5 × 10^9^ CFUs, EcN, EcN-TNFαNb, or EcN-IL10) was administered via gavage every day. **B** Weight change of the mice in each group. **C–D** Colon status (**C**) and length (**D**) of each group of mice. **E** Histological image of colon sections stained with HE (200×). Scale bars, 100 μm. **F** Histology activity index of mice in each group. **G** DAI on the 5th day of DSS administration. **H–I** Analysis of the expression level of anti-TNF-α nanobody (**H**) and IL-10 (**I**) expressed by the engineered bacteria in feces of mice with colitis. The content of anti-TNF-α nanobody and IL-10 in the collected stool samples on the 5th day was detected by ELISA. **J–M** The protein TNF-α (**J**), IL-6 (**K**), IL-17 A (**L**), and MCP-1 (**M**) levels of in the colon samples of mice in each group were determined by ELISA. **N** Masson’s trichrome staining was performed to detect fibrosis, with magnifications of 200× (top) and 400× (bottom). Fibrotic regions are stained blue and indicated by yellow arrows. Scale bars, 100 μm. **O** Collagen proportionate areas in mice from the six groups (mean ± SEM; *n* = 4; individual dots represent individual mice; statistical significance was determined by one-way ANOVA test; *P ≤ 0.05, **P ≤ 0.01, ***P ≤ 0.001, ****P ≤ 0.0001)

As it is known, IBD is notoriously challenging to cure and prone to frequent recurrences, so we have devised a prophylactic strategy aimed at mitigating the risk of recurrence among patients with colitis. The limited gut colonization ability of EcN as a probiotic (Zou et al. 2023) prompted us to administer gavage every 24 h for 7 consecutive days, ensuring continual delivery of engineered bacteria to the intestine. Specifically, mice were orally administered PBS, EcN (containing the α-hemolysin secretion system), EcN-TNFaNb, EcN-IL10, and EcN-TNFαNb + EcN-IL10 daily for 2 days prior to DSS intake and throughout the subsequent 5 days of DSS treatment period (Fig. 3A). As anticipated, both the PBS (DSS^+^) group and the EcN (DSS^+^) group displayed a considerable weight reduction of approximately 10% during the final 2 days of DSS treatment (Fig. 3B). However, mice treated with constitutive engineered bacteria also exhibited a reduction in body weight, potentially attributed to the high expression of drug proteins in constitutive strains, leading to certain toxicity towards the mice. The mice were euthanized on the seventh day for a colon examination, as the colon length serves as an indicator of the severity of inflammation. Notably, the colons of mice in the EcN-TNFαNb + EcN-IL10 (DSS^+^) group displayed a significant increase in length (approximately 1.5 cm) compared to those of diseased mice (PBS DSS^+^) (Fig. 3D), suggesting that the combination of two engineered bacteria mitigated disease progression. In terms of colon condition, DSS^+^ mice treated with EcN-TNFaNb, EcN-IL10, and EcN-TNFαNb + EcN-IL10 demonstrated substantial relief from colon shortening along with reduced areas of congestion and edema (Fig. 3C). Furthermore, when compared to single engineered bacteria treatment, the combination therapy resulted in more complete fecal shape within the colon (Fig. 3C). Compared with the mice given PBS, mice given engineered EcN were lively in spirit and reacted normally to the outside world. Hematochezia and soft stools were alleviated. The HE staining was employed to assess the impact of the engineered bacteria on gut inflammation. Histological imaging of DSS-treated mice (PBS DSS^+^) clearly revealed cell infiltration and epithelial exfoliation, while the EcN-IL10 (DSS^+^) and EcN-TNFαNb + EcN-IL10 (DSS^+^) group exhibited reduced inflammatory cell infiltration and maintained satisfactory colorectal integrity (Fig. 3E). Additionally, a histology activity index was utilized to assess the ulcer count, epithelial cell alterations, and inflammatory infiltration. When compared to the inflammation group (PBS DSS^+^), the group receiving the combination of engineered bacteria exhibited a noteworthy decrease in the activity index score (Fig. 3F). The disease activity index (DAI) of the EcN-TNFαNb + EcN-IL10 (DSS+) group was significantly lower compared to the inflammation group (PBS DSS+) (Fig. 3G). To further investigate the mechanisms behind the observed improvements, the levels of anti-TNF-α nanobody and IL-10 were quantified in mouse feces during DSS-induced colitis. The mice received daily oral administration of EcN, EcN-TNFαNb, EcN-IL10, and EcN-TNFαNb + EcN-IL10 (5 × 10^9^ CFU) for 2 days prior to DSS intake and throughout the 5 days period of DSS intake. Fecal samples were collected from each group of mice on the 7th day. The levels of TNF-α Nb and IL-10 were quantified by ELISA and were determined by subtracting the levels detected in the EcN (DSS^+^) group from those measured in the EcN-TNFαNb, EcN-IL10, and EcN-TNFαNb + EcN-IL10 groups. Our findings revealed that on the 7th day, the concentrations of anti-TNF-α nanobody and IL-10 in the feces of the mice belonging to the EcN-TNFαNb + EcN-IL10 (DSS^+^) group were 822.8 ng/g and 376 ng/g respectively (Fig. 3H–I). To assess the impact of EcN-TNFαNb, EcN-IL10, and EcN-TNFαNb + EcN-IL10 on inflammatory factors, the levels of four cytokines were quantified. Notably, in the PBS (DSS^+^) group treated with EcN-TNFαNb + EcN-IL10, the concentrations of these cytokines were lower than in the PBS (DSS^−^) group (Fig. 3J–M). Moreover, the EcN (DSS+) group exhibited a significant decrease in the levels of the four cytokines in the colon compared to the inflammation group (PBS DSS+). These finding indicated that EcN alone possesses a positive effect on IBD without the need of drug protein intervention. The cytokine levels observed in the EcN-TNFαNb + EcN-IL10 (DSS^+^) group did not significantly differ from those in the EcN (DSS^+^) group. However, treatment with either EcN-TNFαNb or EcN-IL10 alone demonstrated inferior efficacy to combined treatment with both agents (Fig. 3J–M). These results indicated that engineered bacteria expressing anti-TNF-α nanobody and IL-10 are crucial and effective components for treating inflammation alongside the probiotic role of EcN.

The constitutive excretion of drugs at high concentrations may potentially lead to adverse effects, such as fibrosis at the site of injury (Zou et al. 2023). To validate the efficacy of this system in preventing fibrosis formation, collagen proportionate areas were analyzed in different treatment groups using Masson’s trichrome staining of colon sections as a marker for fibrosis. The results demonstrated that the administration of EcN-TNFαNb and EcN-IL10 strains did not significantly exacerbate fibrosis. However, treatment with EcN-TNFαNb resulted in gut fibrosis, indicating an unfavorable safety profile (Fig. 3N–O). Therefore, the combination therapy involving EcN-TNFαNb and EcN-IL10 holds great potential for targeted inflammation treatment.

### Regulatory effects on the gut microbiome

**Fig. 4:**
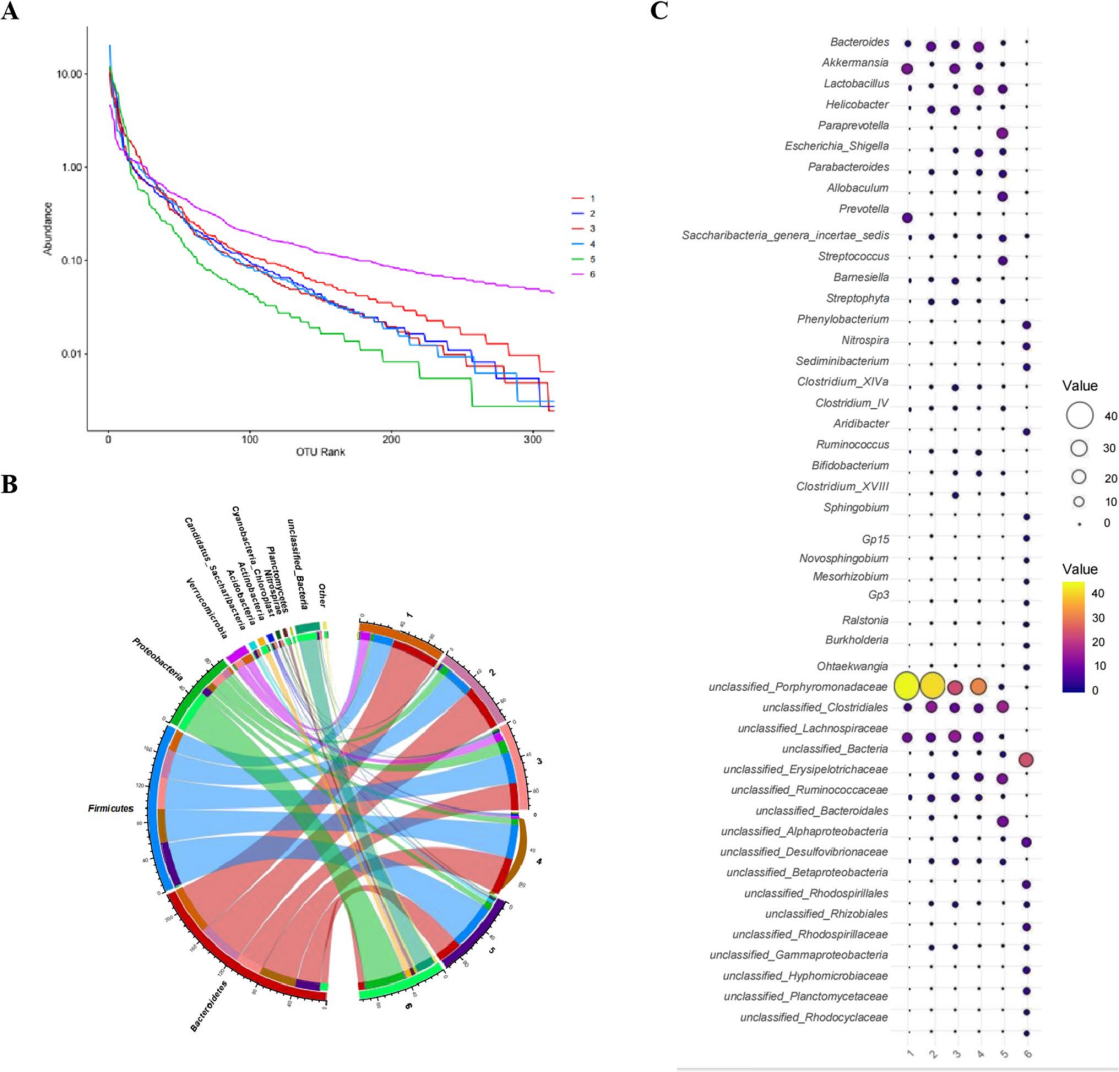
Gut microbiome changes after administration of bacteria in mice with colitis. **A** Rank-abundance curves of species on the operational taxonomic units (OTU). **B** Relative species abundance at the phylum level. **C** Relative species abundance at the genus level. 1, PBS (DSS^−^) (SRR25898823); 2, PBS (DSS^+^) (SRR25898822); 3, EcN (DSS^+^) (SRR25898821); 4, EcN-TNFαNb (DSS^+^) (SRR25898820); 5, EcN-IL10 (DSS^+^) (SRR25898819); 6, EcN-TNFαNb + EcN-IL10 (DSS^+^) (SRR25898818)

We aimed to determine whether the combined use of EcN-TNFαNb and EcN-IL10 could regulate the intestinal microbiota composition in a murine model of DSS-induced inflammatory bowel disease (IBD). Subsequently, we performed 16 S rRNA gene sequencing to assess the abundance of intestinal microbiota. The results from α-diversity analysis revealed that compared with the other five groups, the EcN-TNFαNb + EcN-IL10 group exhibited a more even distribution of microorganisms (Fig. 4A) (https://dataview.ncbi.nlm.nih.gov/object/PRJNA1012370?reviewer=150q5ddlm17fdr3qtrdmt4dfrc). At the phylum level, the relative abundances of *Bacteroidetes* were determined to be 57.24% for the PBS (DSS^−^) group and 52.84% for the PBS (DSS^+^) group, whereas *Firmicutes* accounted for 25.13% in the PBS (DSS^−^) group and 33.23% in the PBS (DSS^+^) group. Notably, *Proteobacteria* exhibited an abundance of 3.08% in the PBS (DSS^−^) group compared with a higher proportion of 9.46% in the PBS (DSS^+^) group. It is worth mentioning that an increased ratio of *Bacteroidetes* to *Firmicutes* has been associated with improved colitis outcomes in mice models. Remarkably, administration of EcN-TNFαNb and EcN-IL10 combination significantly augmented both *Bacteroidetes* and *Firmicutes* proportions, which had been reduced due to DSS treatment (Fig. 4B). Compared to the healthy mice, the genus-level abundances of *Akkermansia* and *Lactobacillus* were significantly reduced in fecal samples from mice with IBD. However, administration of EcN, EcN-TNFαNb, or EcN-IL10 resulted in increased abundances of *Akkermansia*, *Lactobacillus*, and *Bifidobacterium*. The presence of *Akkermansia muciniphila* was found to mitigate colonic histological damage and restore intestinal barrier integrity (Zhai et al. 2019). Furthermore, it has been demonstrated that the tryptophan-metabolizing strain *Lactobacillus reuteri* can prevent colitis in mice (Nikolaus et al. 2017). Members of the *Clostridia*, such as *Clostridium_XlVa* and *Clostridium_IV*, exhibited a reduction in abundance within PBS (DSS^+^) group. *Ruminococcus*, an inflammatory polysaccharide-producing bacterium, demonstrated enrichment in mice with IBD. The abundance of *Ruminococcus* was completely depleted following treatment with a combination of EcN-TNFαNb and EcN-IL10. Furthermore, the presence of *Escherichia-Shigella*, a virulent pathogen known to promote IBD (Feng et al. 2020), decreased after administration of EcN-TNFαNb and EcN-IL10 in DSS-treated mice at the genus level (Fig. 4C). The 16 S rRNA results confirmed that the combined intervention of EcN-TNFαNb and EcN-IL10 modulated the gut microbiome in DSS-induced IBD mice.

### Thiosulfate-responsive genetic circuits for the expression of anti-TNF-α nanobody and IL-10

**Fig. 5:**
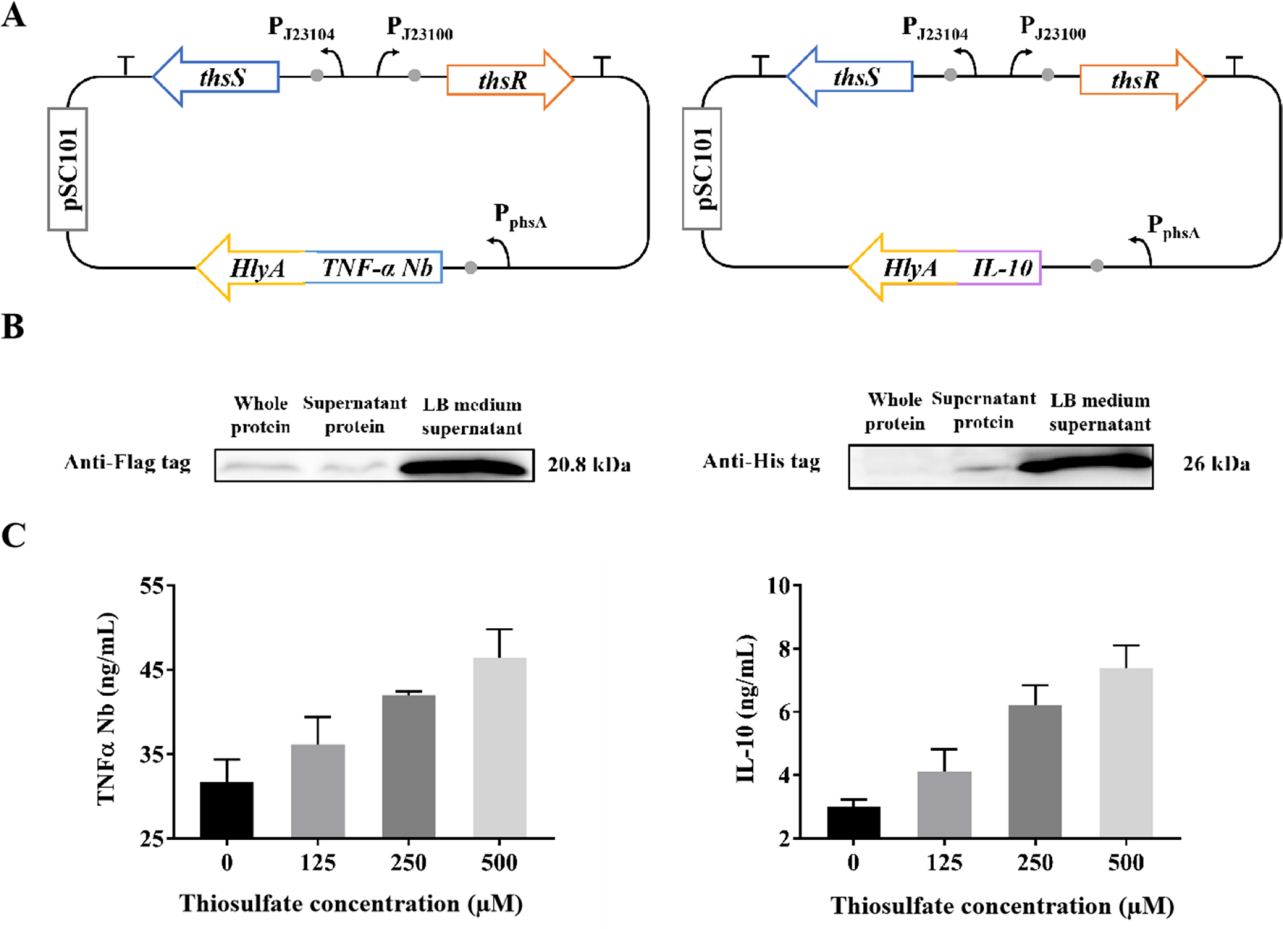
Characterization of inducible engineered bacteria. **A** Gene route design of inducible engineered bacteria thio-EcN-TNF-α Nb (left) and thio-EcN-IL10 (right). **B** Western blot detection results of TNF-α Nb (left) and IL-10 (right) secretion. After 10 h of induction with thiosulfate, the supernatant (“LB medium supernatant”) was collected from the culture by centrifugation, and the pellet was suspended in PBS and then subjected to ultrasonic cell disruption to obtain whole protein from the cells (“whole protein”). Subsequently, the supernatant from the samples (“supernatant protein”) was collected through centrifugation. **C** Detection of TNF-α Nb (left) and IL-10 (right) secretion in culture medium by ELISA

Thiosulfate has demonstrated promising potential as a diagnostic marker for enteritis. In IBD mice, the physiological level of thiosulfate ranges from 35 to 180 µM (Daeffler et al. 2017; Zou et al. 2023). To construct thiosulfate-responsive genetic circuits in EcN, we employed a two-component system, ThsS/R (Supplemental Fig. S4). Initially, we integrated *thsS/R* into a low-copy plasmid under the control of constitutive promoters P_*J23104*_ and P_*J23100*_, incorporating the pSC101 origin of replication to enhance plasmid stability. Additionally, anti-TNF-α nanobody and IL-10 were placed under the regulation of the promoter P_*phsA*_, which responds to thiosulfate via ThsR (Fig. 5A). The integration of drug protein genes did not induce significant toxicity or inhibit strain growth significantly (Supplemental Fig. S5). ELISA and western blotting analysis confirmed detectable levels of anti-TNF-α nanobody and IL-10 in the culture medium of both thio-TNFαNb and thio-IL10 strains. Furthermore, expression levels of anti-TNF-α nanobody and IL-10 exhibited dose-dependent induction by varying concentrations of thiosulfate ranging from 0 to 500 µM (Fig. 5B–C).

## Discussion

The global prevalence of IBD is increasing, imposing a significant burden on patients’ families due to its detrimental effects (Chen et al. 2023). Currently, pharmacotherapy remains the cornerstone in the management of IBD, aiming to disease progression and alleviate symptoms.

The use of engineered live bacterial therapeutics for delivering anti-inflammatory drugs exhibits tremendous potential as an effective treatment approach for IBD. With the increasing availability of genetic tools for microbial engineering, numerous probiotics can be modified and harnessed in IBD therapy (Steidler et al. 2000; Vandenbroucke et al. 2010). In this study, we constructed two engineered strains, EcN-TNFαNb and EcN-IL-10, capable of expressing and secreting anti-TNF-α nanobody and IL-10, respectively. Furthermore, an engineered strain EcN was constructed to secrete TNF-α nanobodies via the type III secretion apparatuses (T3SA) for the treatment of IBD (Lynch et al. 2023). We have thoroughly examined the therapeutic benefits of these strains on IBD through rigorous animal model-based investigations.

First, the ELISA detection results indicated that the anti-TNF-α nanobody exhibited specific binding to mouse tumor necrosis factor-α and this binding is dose-dependent between the antigen (mouse TNF-α) and anti-TNF-α nanobody. The therapeutic proteins, including anti-TNF-α nanobody and/or IL-10, can effectively suppress the release of TNF-α, IL-6, and MCP-1 levels in LPS-stimulated RAW264.7 cells. Our experiments revealed that both drug proteins individually alleviated LPS-induced inflammation in RAW264.7 cells, with the combined administration exhibiting a superior effect compared to either protein alone. Although there were no follow-up studies on macrophages from the animal experiments, we used other indicators, such as body weight, length of colon, the content of pro-inflammatory cytokines, and fibrosis, to demonstrate the therapeutic effect of engineered bacteria on IBD.

ELISA and western blotting indicated that anti-TNF-α nanobody and IL-10 could be detected in the medium supernatant of the EcN-TNFαNb and EcN-IL10 strains, and the expression of anti-TNF-α nanobody and IL-10 was time-dependent, ranging from 6 to 12 h. We subsequently assessed the therapeutic efficacy of the engineered bacteria alone and in combination with the dual bacterial treatment. EcN-TNFaNb and EcN-IL10 demonstrated effective mitigation of DSS-induced mouse colitis, as indicated by reduced colon length shortening, lower disease activity index (DAI), and altered expression of pro-inflammatory cytokines in colon tissues, such as TNF-α, IL-6, IL-17 A, and MCP-1. More interestingly, architectural changes, inflammatory cell infiltrations, and epithelial injuries were reduced in the EcN-TNFaNb or/and EcN-IL10 group by hematoxylin and eosin (HE) staining. Animals given EcN-IL10 stopped losing weight after 3 days, which may be due to changes in food intake. However, in this study, we did not measure food intake among the different groups over the entire course of the experiment. This needs to be addressed in further experiments. In addition, all DSS + animals showed a decrease in *Bifidobacterium*, *Akkermansia*, and *Lactobacillus* compared with DSS^**−**^ animals (Fig. 4B), because colitis induced by DSS in mice reduces the levels of *Firmicutes*, *Actinobacteria*, and others (Xu et al. 2021). However, compared with the PBS DSS + group, administration of EcN, EcN-TNFαNb, or EcN-IL10 resulted in increased abundances of *Bifidobacterium*, *Akkermansia*, and *Lactobacillus*. Moreover, the increasing ratio of *Bacteroidetes* to *Firmicutes* has been associated with improved colitis outcomes in mice models (Li et al. 2022), and administration of EcN-TNFαNb and EcN-IL10 combination significantly augmented the ratio of *Bacteroidetes* to *Firmicutes*, which had been reduced due to DSS treatment.

These results mean that the administration of the engineered bacteria has a certain regulatory effect on IBD. Although our work provides preliminary evidence of the effects of engineered bacteria on the gut microbiota of enteritis mice, further in-depth research is necessary to uncover the underlying mechanisms in the future work. The efficacy of IBD treatment in mice is enhanced with a combination of dual bacterial strains.

After conducting experiments on mice with constitutive engineered bacteria, it has been established that the high toxicity of these bacteria may lead to additional inflammation. Furthermore, we have successfully engineered inducible genetically modified bacteria that possess the capability to regulate the secretion levels of drug proteins. ELISA and Western blotting revealed that the expression of anti-TNF-α nanobody and IL-10 were induced by thiosulfate in a dose-dependent manner from 0 to 500 µM, which is the range of intestinal thiosulfate concentrations in DSS-induced inflammatory bowel disease in mice according to previous studies (Zou et al. 2023).

The microorganism engineered with intelligence in the current experiments comprises solely of sensing and drug-releasing elements, yet its capabilities can be further expanded. Henceforth, the focal point of this study will be on the exploration of efficient methods for the secretion of multiple proteins within a singular bacterial cell in the upcoming future.

Previous works had proven that TNF-α nanobodies (Vandenbroucke et al. 2010) and IL-10 exhibited satisfying effects on the treatment of IBD (Steidler et al. 2000). Moreover, the level of IL-10 in people with enteritis was increased, which could effectively inhibit inflammation (Kucharzik et al. 1995; Wang et al. 2011). Notably, the effects of IL-10 expressed by an engineered bacterial strain was much better than that of purified IL-10 in other reports (Steidler et al. 2000). In this study, our main focus was on investigating the alleviating effect of using engineered bacteria (single engineered bacteria or cocktails) as carriers to express and deliver therapeutic proteins for inflammatory bowel disease. Therefore, we did not include a control group of animals that received purified TNF-a nanobodies and IL-10 intravenously in this study. Although the modified engineered bacteria have demonstrated efficacy in treating IBD, several issues persist. First, it remains unclear whether these bacteria can replicate their success in other animal models beyond mice. Second, our engineered bacteria require further research to determine their stability and safety in relation to human health. Thirdly, unlike chemotherapy drugs and biopharmaceutical agents, bacterial-based drug delivery systems cannot undergo conventional sterilization methods, rendering the establishment of good manufacturing practice (GMP) more challenging.

Despite the existing uncertainties, it is undeniable that in vivo drugs based on synthetic biology technology are highly customizable and align with the general direction of precision medicine. Their development prospects are promising, and we anticipate that more patients suffering from IBD will benefit from this study in the future.

## Supplementary Information

Below is the link to the electronic supplementary material.

Supplementary Material 1
