# Supplementary material for: Dual engineered bacteria improve inflammatory bowel disease in mice
Source: Appl Microbiol Biotechnol. 2024 May 13;108:333. doi: 10.1007/s00253-024-13163-w (PMC11090975; doi:10.1007/s00253-024-13163-w)
Supplement: Supplementary file 1 [file 253_2024_13163_MOESM1_ESM.pdf]

Suppl. Material

Journal: Applied Microbiology and Biotechnology

Dual engineered bacteria improve inflammatory bowel disease in mice

Yong-Qi Wu[a, #], Zhen-Ping Zou[a, #], Ying Zhou[a] *, and Bang-Ce Ye[a,b] *

[a] *Laboratory of Biosystems and Microanalysis, State Key Laboratory of Bioreactor Engineering, East China University of Science and Technology, Shanghai 200237, China*

[b] *Institute of Engineering Biology and Health, Collaborative Innovation Center of Yangtze River Delta Region Green Pharmaceuticals, College of Pharmaceutical Sciences, Zhejiang University of Technology, Hangzhou 310014, Zhejiang, China*

[#] *Y.-Q. W. and Z.-P. Z. contributed equally to this paper*

[*] Email: zhouying@ecust.edu.cn (Y.Z.); bcye@ecust.edu.cn (B.-C. Y.)

Suppl. Figures

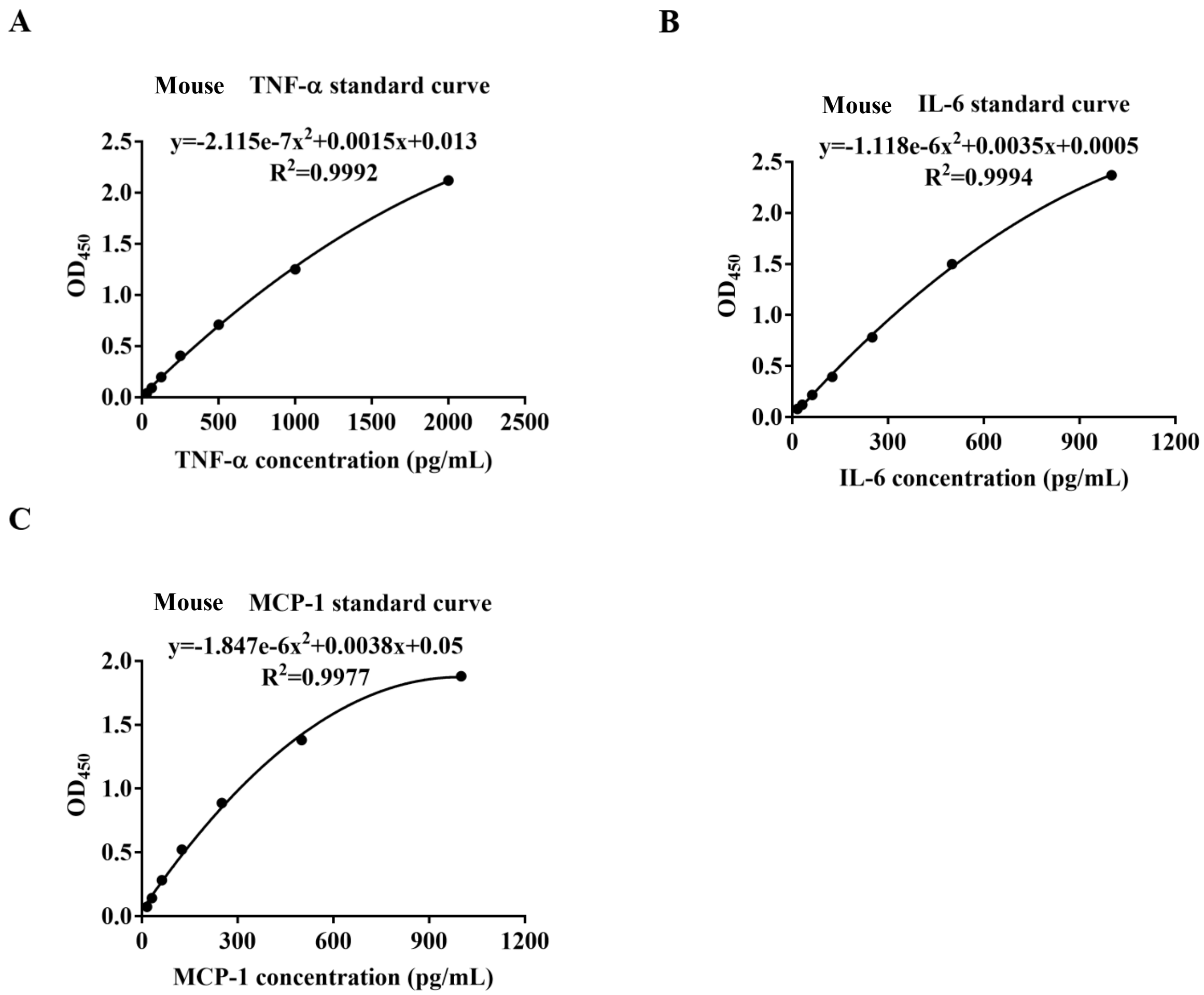

Supplemental Fig. S1 Mouse TNF-α standard curve (A); Mouse IL-6 standard curve (B); Mouse MCP-1 standard curve (C).

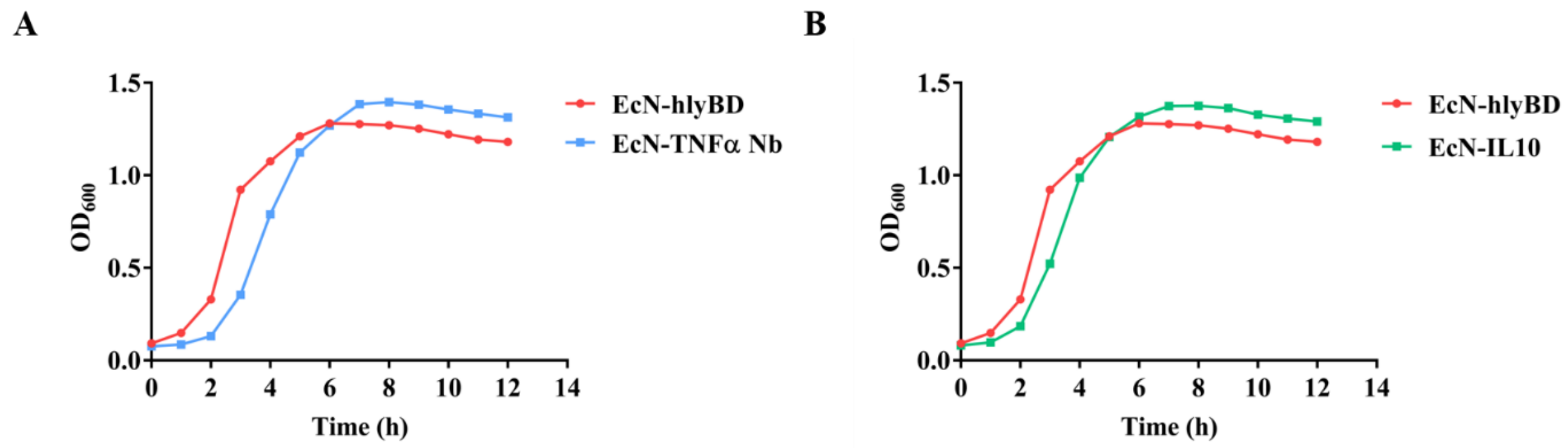

Supplemental Fig. S2 Growth curve of constitutive engineered bacteria EcN-TNF-α Nb (A) and EcN-IL10 (B).

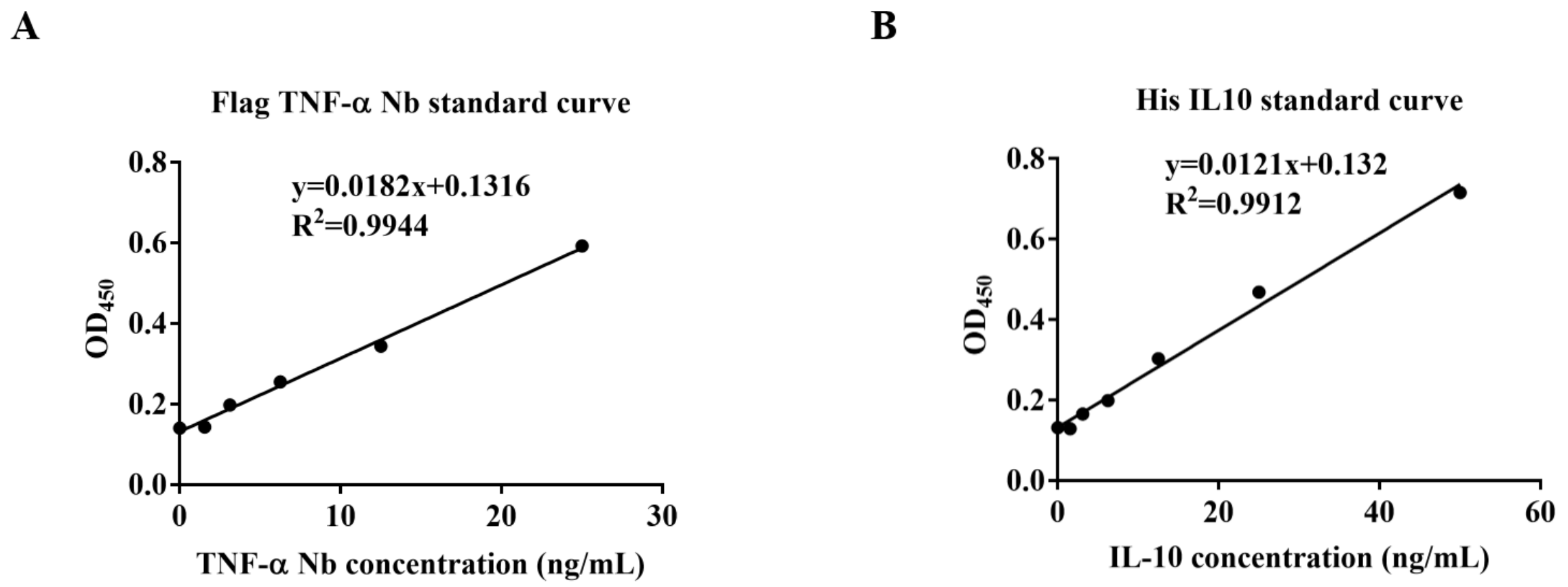

Supplemental Fig. S3 Standard curve of anti-TNF-α nanobody (A) and IL-10 (B).

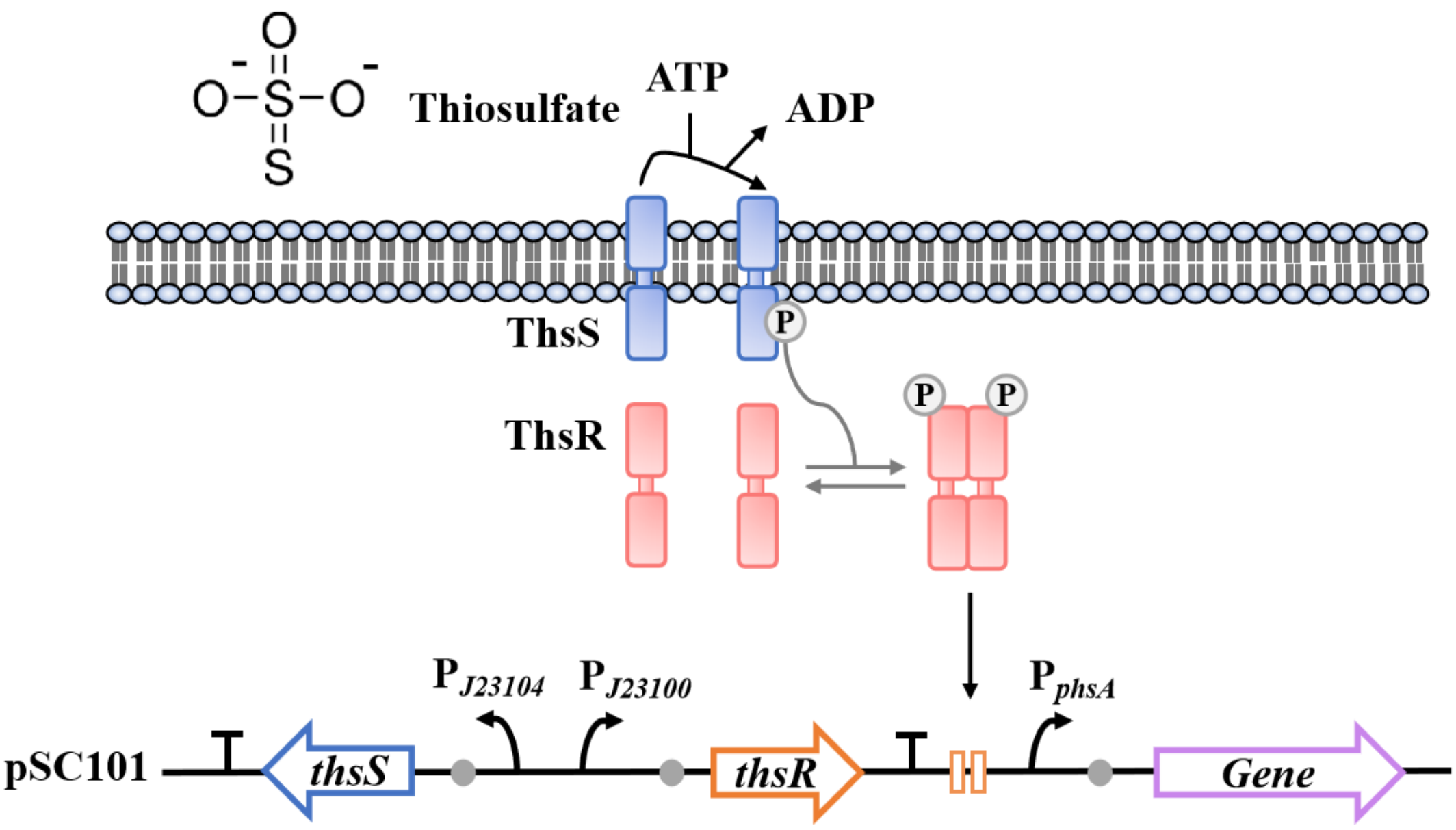

Supplemental Fig. S4 Schematic diagram of the thiosulfate two-component system (Daeffler et al. 2017)

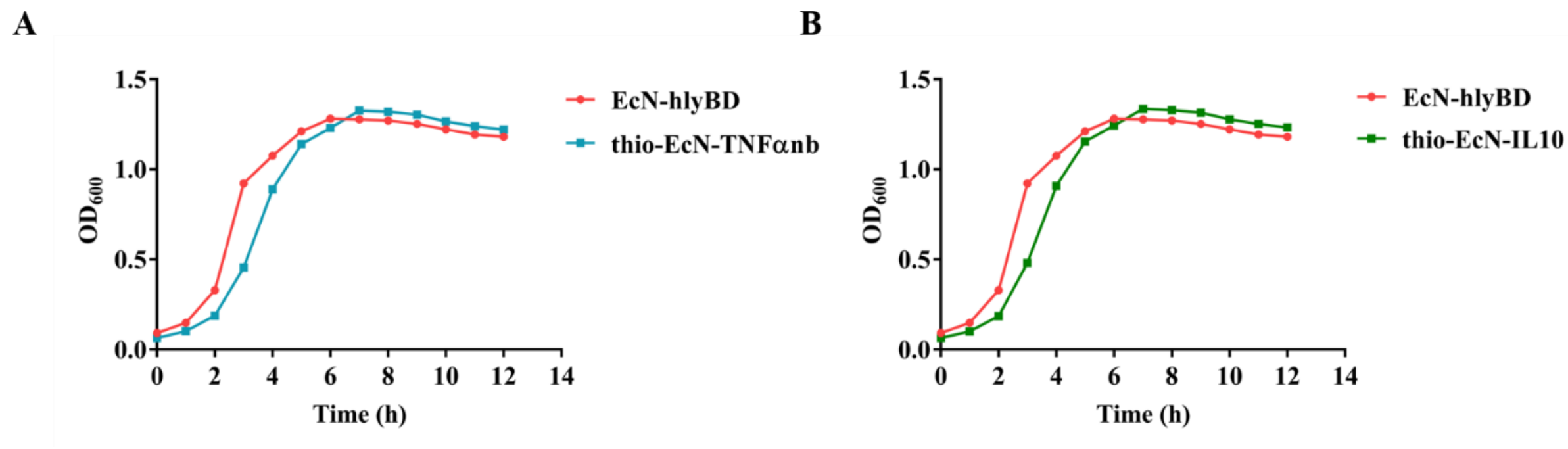

Supplemental Fig. S5 Growth curve of inducible engineered bacteria thio-EcN-TNF-α Nb (A) and thio-EcN-IL10 (B).

Suppl. Tables

Supplemental Table S1 Strains and plasmids used in this work.

Strain/Plasmid	References/Sources
Escherichia coli DH5α	TransGen Biotech, Beijing, China
Escherichia coli BL21(DE3)	TransGen Biotech, Beijing, China
pET28a-TNFα Nb-BL21	This study
pET28a-IL10-BL21	This study
Escherichia coli Nissle 1917 (EcN)	DSMZ Cat# DSM6601
EcN-hlyBD	(Zou et al. 2023)
EcN-mcherry	This study
pWT-TNFaNb	This study
EcN-TNFα Nb	This study
pWT-IL10	This study
EcN-IL10	This study
thio-EcN-TNFα Nb	This study
thio-EcN-IL10	This study

Supplemental Table S2 Overview of all sequences of genetic parts used in this work.

Gene/encoded protein	Nucleotide sequences	Amino acid sequences
Anti-TNF-α antibody	CAGGTGCAGCTGCAAGATAGCGGTGGTGGTCTGGTTCAGG<br>CAGGTGGTAGTCTGCGCCTGAGTTGCGCAGCCAGTGGCGG<br>TACATTTAGTAGCATTATTATGGCCTGGTTCCGTCAGGCACC<br>GGGCAAAGAACGTGAATTTGTGGGCGCAGTGAGCTGGAGT<br>GGTGGCACCACCGTTTATGCAGATAGTGTTCTGGGTCGTTT<br>TGAAATTAGCCGCGATAGTGCCCGTAAAAGTGTGTATCTGC<br>AAATGAATAGCCTGAAACCGGAAGATACCGCCGTTTATTATT<br>GCGCAGCCCGCCCGTATCAGAAATATAATTGGGCCAGCGC<br>AAGCTATAATGTGTGGGGCCAGGGCACCCAGGTGACCGTT<br>AGTAGT	QVQLQDSGGGLVQAGGSLRLSCAASGGTFSSII<br>MAWFRQAPGKEREFVGAVSWSGGTTVYADSVL<br>GRFEISRDSARKSVYLQMNSLKPEDTAVYYCAAR<br>PYQKYNWASASYNVWGQGTQVTVSS
IL-10	AGCCGTGGCCAGTATAGTCGTGAAGATAATAATTGCACCCA<br>CTTTCCGGTGGGTCAGAGCCACATGCTGCTGGAACTGCGC<br>ACCGCATTTAGCCAGGTGAAAACCTTTTTTCAGACCAAAGAT<br>CAGCTGGATAACATTCTGCTGACCGATAGTCTGATGCAGGA<br>TTTTAAAGGTTACCTGGGCTGCCAGGCACTGAGTGAAATGA<br>TTCAGTTTTATCTGGTGGAGGTGATGCCGCAGGCAGAAAAA<br>CATGGCCCGGAAATTAAAGAACATCTGAATAGTCTGGGCGA<br>AAAACTGAAAACCCTGCGCATGCGCCTGCGTCGCTGTCATC<br>GTTTTCTGCCGTGTGAAAATAAAAGCAAAGCCGTTGAACAG<br>GTTAAGAGCGATTTTAATAAGCTGCAGGATCAGGGTGTTTAT	MSRGQYSREDNNCTHFPVGQSHMLLELRTAFS<br>QVKTFFQTKDQLDNILLTDSLMQDFKGYLGCQAL<br>SEMIQFYLVEVMPQAEKHGPEIKEHLNSLGEKLK<br>TLRMRLRRCHRFLPCENKSKAVEQVKSDFNKLQ<br>DQGVYKAMNEFDIFINCIEAYMMIKMKS

	AAAGCCATGAATGAATTTGACATCTTCATCAACTGCATCGAAGCCTATATGATGATTAAGATGAAGTCC	
mcherry	ATGGTGAGCAAGGGCGAGGAGGATAACATGGCCATCATCAAGGAGTTCATGCGCTTCAAGGTGCACATGGAGGGCTCCGTGAACGGCCACGAGTTCGAGATCGAGGGCGAGGGCGAGGGCCGCCCCTACGAGGGCACCCAGACCGCCAAGCTGAAGGTGACCAAGGGTGGCCCCCTGCCCTTCGCCTGGGACATCCTGTCCCCTCAGTTCATGTACGGCTCCAAGGCCTACGTGAAGCACCCCGCCGACATCCCCGACTACTTGAAGCTGTCCTTCCCCGAGGGCTTCAAGTGGGAGCGCGTGATGAACTTCGAGGACGGCGGCGTGGTGACCGTGACCCAGGACTCCTCCCTGCAGGACGGCGAGTTCATCTACAAGGTGAAGCTGCGCGGCACCAACTTCCCCTCCGACGGCCCCGTAATGCAGAAGAAGACCATGGGCTGGGAGGCCTCCTCCGAGCGGATGTACCCCGAGGACGGCGCCCTGAAGGGCGAGATCAAGCAGAGGCTGAAGCTGAAGGACGGCGGCCACTACGACGCTGAGGTCAAGACCACCTACAAGGCCAAGAAGCCCGTGCAGCTGCCCGGCGCCTACAACGTCAACATCAAGTTGGACATCACCTCCCACAACGAGGACTACACCATCGTGGAACAGTACGAACGCGCCGAGGGCCGCCACTCCACCGGCGGCATGGACGAGCTGTACAAG	MVSKGEEDNMAIIKEFMRFKVHMEGSVNGHEFEIEGEGEGRPYEGTQTAKLKVTKGGPLPFAWDILSPQFMYGSKAYVKHPADIPDYLKLSFPEGFKWERVMNFEDGGVVTVTQDSSLQDGEFIYKVKLRGTNFPSDGPVMQKKTMGWEASSERMYPEDGALKGEIKQRLKLKDGGHYDAEVKTTYKAKKPVQLPGAYNVNIKLDITSHNEDYTIVEQYERAEGRHSTGGMDELYK
hlyA secretory peptide	TTAGCCTATGGAAGTCAGGATAATCTTAATCCATTAATTAATGAAATCAGCAAAATCATTTCAGCTGCAGGTAATTTTGATGTTAAAGAGGAAAGAGCTGCAGCTTCTTTATTGCAGTTGTCCGGTAATGCCAGTGATTTTTCATATGGACGGAACTCAATAACTTTGACAGCATCAGCA	LAYGSQDNLNPLINEISKIISAAGNFDVKEERAAASLLQLSGNASDFSYGRNSITLTASA

References

Daeffler KN, Galley JD, Sheth RU, Ortiz-Velez LC, Bibb CO, Shroyer NF, Britton RA, Tabor JJ (2017) Engineering bacterial thiosulfate and tetrathionate sensors for detecting gut inflammation. Mol Syst Biol 13: 923. https://doi.org/10.15252/msb.20167416

Zou ZP, Du Y, Fang TT, Zhou Y, Ye BC (2023) Biomarker-responsive engineered probiotic diagnoses, records, and ameliorates inflammatory bowel disease in mice. Cell Host Microbe 31: 199-212. https://doi.org/10.1016/j.chom.2022.12.004
